# A Joint Technology Combining the Advantages of Capillary Microsampling with Mass Spectrometry Applied to the Trans-Resveratrol Pharmacokinetic Study in Mice

**DOI:** 10.1155/2022/5952436

**Published:** 2022-01-17

**Authors:** Ying Xu, Song-xia Zhang, Jing Guo, Li-jie Chen, Yu-ligh Liou, Tai Rao, Jing-bo Peng, Ying Guo, Wei-hua Huang, Zhi-rong Tan, Dong-sheng Ou-yang, Hong-hao Zhou, Wei Zhang, Yao Chen

**Affiliations:** ^1^Department of Clinical Pharmacology, Xiangya Hospital, Central South University, Changsha, Hunan, China; ^2^Institute of Clinical Pharmacology, Central South University, Changsha, Hunan, China; ^3^Engineering Research Center of Applied Technology of Pharmacogenomics, Ministry of Education, Changsha, Hunan, China; ^4^National Clinical Research Center for Geriatric Disorders, Changsha, Hunan, China

## Abstract

Mice are the most frequently used animals in pharmacokinetic studies; however, collecting series of blood samples from mice is difficult because of their small sizes and tiny vessels. In addition, due to the small sample size, it is problematic to perform high required quantification. Thus, present work aims to find an effective strategy for overcoming these challenges using trans-resveratrol as a tool drug. Based on the idea of a joint technology, the capillary microsampling (CMS) was chosen for blood sample collection from mice after delivery of trans-resveratrol (150 mg/kg) by gavage, and a high-performance liquid chromatography-tandem mass spectrometry (HPLC-MS/MS) method was developed for the determination of trans-resveratrol and its main metabolites. All the mouse blood samples were exactly collected by CMS without obvious deviation. This provided credible samples for subsequent quantitative analysis. The HPLC-MS/MS method was found to be sensitive, accurate, and repeatable, and the pharmacokinetic parameters for all analytes were comparable with those reported in previous studies. However, the present joint technology offers the advantages of less animal damage, easy for sample preparation, and improved reliability. It has overcome some of the major limitations revealed in previous pharmacokinetic studies in mice and therefore provides a more effective option for future studies.

## 1. Introduction

Preclinical pharmacokinetic studies, including single- or multiple-dose toxicology pharmacokinetic studies, conducted in animals have been considered a key approach for obtaining toxicological data for a new compound. According to the Foundation for Biomedical Research [1] and the American Association for Laboratory Animal Science [2], mice are frequently used in this type of experimental research. A pharmacokinetic study usually requires several biological samples, such as blood, serum, and plasma, from the experimental animals at different series of time points [3, 4]. When mice are used, their small size poses difficulties. The accurate penetration of the blood collection needle into the tiny blood vessels presents one of the greatest difficulties for series of blood sampling. In addition, the blood volume for each time point collection is not large because of the small body weight of the mice. Therefore, mice inherent characteristics pose challenges for sampling although they are the most often used mammals in preclinical pharmacokinetic studies.

Over the past decades, microsampling techniques have been developed and extensively used in medical studies. Because of the advantages of reduction, refinement, and replacement (3R principles) [5], capillary microsampling (CMS) [6], volumetric absorptive microsampling (VAMS) [7], and dried blood spot sampling (DBS) [8, 9] have been the most used techniques. CMS was recently introduced in response to the demands for the more ethical use of laboratory animals in accordance with the 3R principles. With CMS, the exact volume of the blood or other biofluids is collected into the capillaries and then used for different purposes including qualitative or quantitative determination. For VAMS, the difference with CMS is that it requires the use of special auxiliary devices, which obviously increases the cost. DBS is the collection of whole blood with filter paper, and dried biological samples can be conveniently kept and shipped. It seems more suitable for the qualitative examination of diseases but offers no more advantages over CMS for quantitative determination [10]. Therefore, CMS was the optimal choice for blood sampling from mice in the present study.

While CMS is a basic traditional microsampling technique, its application in mice pharmacokinetic studies is not universal. One of the major reasons might be the stringent requirements for the sensitivity of the quantitation instruments in pharmacokinetic studies because of the very small sample volumes obtained from mice until the rapid progress of mass spectrometry. To date, high-performance liquid chromatography-tandem mass spectrometry (HPLC-MS/MS) has been able to detect most compounds at very low concentrations. By improving the sensitivity, HPLC-MS/MS could significantly reduce the consumption of sample volume during the detection process. It provides a good detection technology platform for the study of pharmacokinetics in mice.

As mentioned above, mice play a major role in preclinical pharmacokinetic studies; however, the aforementioned challenges have seriously limited the practical application. CMS could solve the problem of insufficient sample quantities collected from a little mouse, and the high sensitivity of HPLC-MS/MS could compensate for the disadvantages of CMS for collecting very small sample volumes per time point. Therefore, a strategy that combines the advantages of CMS with the high sensitivity of HPLC-MS/MS might solve the problems in mice pharmacokinetic studies. The present study aimed to determine the feasibility of this joint technology for overcoming those challenges using trans-resveratrol just as a tool compound.

## 2. Experimental

### 2.1. Chemicals and Reagents

**Figure 1:**
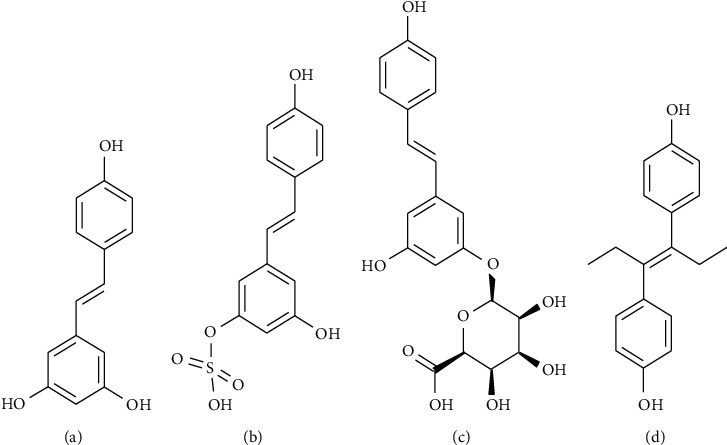
The molecular structures for trans-resveratrol (a), trans-resveratrol-3-sulfate salt (b), trans-resveratrol-3-o-*β*-glucuronide (c), and diethylstilbestrol (d).

Glass capillaries (40 *μ*L, coated with heparin sodium) were purchased from the Zibo Laixu Medical Equipment Co. Ltd. (Shandong, China). Mouse fixators were purchased from Zhongke Life Science & Technology Co. Ltd. (Hangzhou, China). Trans-resveratrol (purity: 99%, Lot No. E1711079) was purchased from Aladdin Industrial Corporation (Shanghai, China). Trans-resveratrol-3-o-*β*-d-glucuronide (purity: 97.23%, Lot No. 6-LXS-20-2) and trans-resveratrol-3-sulfate sodium salt (purity: 96.76%, Lot No. 10-UPA-21-2) were purchased from Toronto Research Chemicals (Brisbane Road, Toronto, Canada). Diethylstilbestrol (purity: 99.9%, Lot No. 100033-200607), which was used as the internal standard (IS), was purchased from the National Institute for Food and Drug Control (Beijing, China). The chemical structures of trans-resveratrol, its main metabolites, and IS are provided in Figure 1. Acetonitrile (HPLC grade) and methanol (HPLC grade) were purchased from Merck (Darmstadt, Germany). Ammonium acetate (HPLC grade, Lot No. V097k090) was obtained from ANPEL Laboratory Technologies (Shanghai, China), and deionized water was obtained from a Millipore Milli-Q Plus system (Bedford, USA).

### 2.2. CMS Applied to the Pharmacokinetic Study of Trans-Resveratrol in Mice

C57BL6 mice were obtained from the Department of Laboratory Animals, Central South University (Changsha, China). All of the experimental animal protocols conformed to the Institutional Animal Ethics Committee (IAEC) of Central South University and the National Institute of Health Guide for the Care and Use of Laboratory Animals. The mice were housed in the Central South University Department of Laboratory Animals in a specific pathogen-free (SPF) animal center. The temperature was controlled at 22 ± 2°C, with the relative humidity set to 60 ± 5%. The days and nights were alternated at 12-h cycles, and the animals had free access to food and water.

Six male C57BL6 mice (6–8 weeks old, 22–26 g) were selected for the subsequent trans-resveratrol pharmacokinetic study. All of the mice were fasted for 12 h before the experiment. Next, trans-resveratrol (150 mg/kg) was delivered to the mice by gavage in a dose that had been previously reported in mice and rats [11, 12]. Glass microcapillaries were then used to collect a series of blood samples (about 15 *μ*L volume each point) at 0, 0.25, 0.5, 0.75, 1, 2, 4, 6, 8, and 12 h from each mouse.

The CMS method is basically referred to those in reported research works with some modifications. The following are the details of the steps for present CMS. Each mouse for blood collection was temporarily placed in a fixator with just its tail exposed. The tail was sterilized with alcohol (75%), and 1–2 mm was cut quickly from the tip with a pair of sharp surgical scissors. The tail was gently pressed from the root to the end to enable to form a drop of blood. The blood sample was collected in a glass microcapillary through capillary action, and then blew it out to a 1.5 mL EDTA-K2 polythene tube with a rubber suction bulb and protect from light immediately. Next, sterilized cotton was pressed directly on the tail wound for approximately 10 seconds to stop the bleeding. Once the collection of the samples for one time point had been completed, the mouse could resume free movement. Food and water were available to the mice 4 h after the delivery of trans-resveratrol. At the next collection time point, just the wound clot was removed with sterilized cotton. The sample was collected, and bleeding was stopped in accordance with the previously described procedures. The cycles were repeated until the last blood sample was collected. The samples were maintained in a −80°C refrigerator away from light for subsequent HPLC-MS/MS analysis.

### 2.3. HPLC-MS/MS Method for the Determination of Trans-Resveratrol and Its Metabolites

A Triple Quad^TM^ 6500 HPLC-MS/MS system (AB Sciex, Concord, Ontario, Canada) equipped with a binary pump (LC-30AD, Shimadzu, Japan), an autosampler (SIL-30AC, Shimadzu, Japan), a degassing unit (DGU-20A5R, Shimadzu, Japan), a communications bus module (CBM-20A, Shimadzu, Japan), and a prominence column oven (CTO-20AC, Shimadzu, Japan) were used in the study. An ACE Excel 5 Super C18 column (50 mm × 2.1 mm, 5 *μ*m, Batch No. V17-1613; ACE, USA) with the column oven temperature set at 40°C and a mobile phase consisting of 5 mM ammonium acetate (A) and acetonitrile (B) set at a flow rate of 0.30 mL/min were used for the separation of the trans-resveratrol, its metabolites, and the IS.

**Figure 2:**
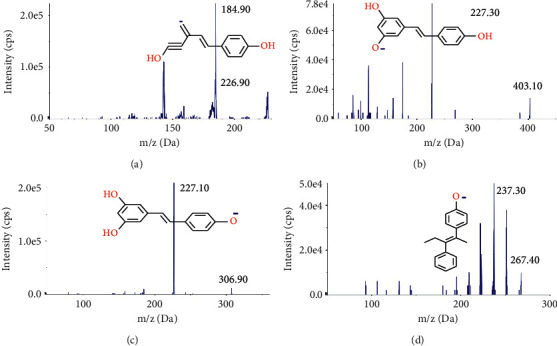
Product ion mass spectra of (a) trans-resveratrol (m/z 226.90–184.90), (b) trans-resveratrol-3-o-*β*-glucuronide (R3G, m/z 403.10–227.30), (c) trans-resveratrol-3-sulfate salt (R3S, m/z 306.90–227.10), and (d) diethylstilbestrol (IS, m/z 267.40–237.30) in negative ionization mode.

The gradient program of the mobile phase was as follows: 0–0.1 min, 10% B; 0.1–3.5 min, 10–100% B; 3.5–3.9 min, 100% B; and 3.9–4.0 min, 100–10% B. This was followed by re-equilibration at 10% B for 5.0 min. The autosampler temperature was maintained at 15°C, and the injection volume was set at 10 *μ*L. The mass spectrometer was operated in a negative ion mode through the use of multiple-reaction monitoring (MRM). The precursor-product ion transitions were monitored at m/z 226.90–184.90 for trans-resveratrol, m/z 403.10–227.30 for trans-resveratrol-3-o-*β*-glucuronide (R3G), m/z 306.90–227.10 for trans-resveratrol-3-sulfate salt (R3S), and m/z 267.40–237.70 for the IS (Figures 2(a)–2(d)). Data acquisition was performed with Analyst Software, version 1.4.2 (AB Sciex, Concord, Ontario, Canada).

### 2.4. Calibration Standards and Quality Control Sample Preparation

The standard stock solutions of trans-resveratrol, R3G, and R3S were prepared with methanol and water (50⁄50, v⁄v) and stored at −80°C. The working calibration solutions were prepared by diluting mixed stands of stock solution with the methanol and water in the aforementioned proportions. Next, a series of concentrations of working calibration solutions (22.35–2235 ng/mL for the trans-resveratrol and 10–2000 ng/mL for the R3G and R3S) were obtained. Calibration-standard samples (2.235–223.5 ng/mL for the trans-resveratrol and 1–200 ng/mL for the R3G and R3S) were produced by spiking 9 *μ*L blank mouse blood with 1 *μ*L mixed working standard solution one by one.

Quality control (QC) working mixed solutions were prepared independently at four different concentration levels: 1117.5, 223.5, 55.88, and 22.35 ng/mL for the trans-resveratrol and 1000, 200, 50, and 10 ng/mL for the R3G and R3S. Four QC samples in different concentration levels (111.75, 22.35, 5.59, and 2.24 ng/mL for the trans-resveratrol and 100, 20, 5, and 1 ng/mL for the R3G and R3S) were also produced by spiking 9 *μ*L blank mouse blood with 1 *μ*L QC working mixed solution corresponding to high (H) QC, medium (M) QC, low (L) QC, and lower limit of quantification (LLOQ) samples individually.

A working diethylstilbestrol (IS) solution was prepared by diluting the IS stock solution with methanol to the set concentration (5.48 ng/mL) and stored at 4°C before use.

### 2.5. Blood Sample Preparation

The mouse blood samples (10 *μ*L) were transferred to 1.5 mL disposable centrifugal tubes (protected from light), mixed with 200 *μ*L methanol containing the IS, which was vortex-mixed for 5 min and centrifuged at 20800 g (4°C) for 10 min to remove the precipitated proteins. Next, 100 *μ*L supernatant was transferred to another clean tube to which 100 *μ*L mobile phase A had been pre-added. The mixture was vortexed for 2 min and re-centrifuged at 20800 g (4°C) for another 5 min. Finally, the 100 *μ*L supernatant was transferred to a sample bottle for HPLC-MS/MS detection.

### 2.6. HPLC-MS/MS Method Validation

HPLC-MS/MS method validation was performed in accordance with the United States Food and Drug Administration (FDA) guidelines [13]. The validation items exhibited selectivity, linearity, LLOQ, accuracy, precision, the matrix effect, recovery, and stability.

#### 2.6.1. Selectivity

Six different mouse blank blood samples were prepared in accordance with the sample preparation and detected by the present HPLC-MS/MS method. The pass requirements, i.e., the peak areas of interference, were less than 20% of the LLOQ. For the IS, the response in the blank samples should not exceed 5% of the average IS response in the calibrator and QC samples.

#### 2.6.2. Linearity and Lower Limit of Quantification

Linearity was validated over three consecutive days through the use of the calibration-standard samples (2.24–223.5 ng/mL for the trans-resveratrol and 1–200 ng/mL for the R3G and R3S), which were analyzed by the present method after sample preparation. The calibration curves were drawn by calibrating the peak area ratio (*y*) of the trans-resveratrol or its metabolites to the IS with the concentration (*x*) of the standard sample. The regression parameters were calculated from the linear least squares regression (1⁄x^2^). The LLOQ was the lowest concentration, with a coefficient of variation (CV) precision of no more than 20%, accuracy 80–120%, and signal-to-noise ratio (S⁄N) no less than 10.

#### 2.6.3. Accuracy and Precision

Intrabatch accuracy and precision were evaluated through six repeated measurements of the same batch of the LQC, MQC, and HQC samples. Interbatch accuracy and precision were evaluated for three batch measurements over three days on the basis of the level of each QC sample. Neither the deviation for accuracy nor the percent CV for precision exceeded ±15%.

#### 2.6.4. Matrix Effect and Recovery

The matrix effect and recovery were performed with three concentrations (low, medium, and high) of analytes from the same batch. The criterion for the matrix effect was 80–120%. The recovery value is usually at least 50%; however, the guidelines are not specific.

#### 2.6.5. Stability

The stability of the trans-resveratrol and its metabolites in the mouse blood was evaluated under the different storage conditions described below. Triplicates of LQC and HQC samples were used. The test conditions included freeze-thaw stability after three freeze-thaw cycles (−80°C to 25°C), short-term stability at room temperature for 4 h, long-term stability at −80°C for 30 days, and autosampler stability at 15°C for 12 h. The concentrations for the QC samples were calculated by comparing the calibration curves of the freshly prepared standards with those of the samples with nominal values. The stability of the trans-resveratrol and its metabolite stock solutions was also determined at 4°C for 30 days. The mean peak areas of three replicates of the trans-resveratrol and its metabolites were compared with those from the solutions that were freshly prepared at the same concentration.

### 2.7. Application and Statistical Analysis

Present joint technology combining the advantages of CMS with HPLC-MS/MS was finally applied to the trans-resveratrol pharmacokinetic study in mice. The pharmacokinetic parameters of the trans-resveratrol and its main metabolites were analyzed by the noncompartmental assessment of the data with Drug and Statistical Software, version 3.2.2 (Clinical Drug Evaluation Center, Wannan Medical College, Anhui, China). The maximum plasma concentration (*C*_max_) and the time of occurrence (*T*_max_) were noted directly from the measured data. The elimination rate constant (*k*_*e*_) was calculated by the log-linear regression of the concentrations observed during the terminal phase of elimination, and the elimination half-life (t1⁄2) was then calculated as 0.693/*k*_*e*_. The area under the plasma concentration-time curve (AUC_0–t_) to the last measurable plasma concentration (C_t_) was calculated with the linear trapezoidal rule. The area under the plasma concentration-time curve to time infinity (AUC_0–∞_) was calculated as AUC_0–t_ + C_t_⁄*k*_*e*_.

## 3. Results and Discussion

### 3.1. Capillary Microsampling

The modified CMS technique was successfully established, and the designed and actual blood collection times are presented in Table 1. The coefficient of variation of the collection times was less than 15%. The results reflect the just-in-time collection of all of the blood samples on the basis of each time point by CMS without obvious deviation.

Blood sampling from mice is a recognized difficulty during pharmacokinetic studies because of their small sizes and tiny vessels. The present CMS is modified on the base of previous CMS methods, and the modification lies on the usage of glass capillary cooperated with just-once-cut tail snipping, which could realize series of blood sampling smoothly for pharmacokinetic studies. We found the blood sampling could be continued for the next collection time point at the same site through removing the wound clot with sterilized cotton, and no more cut-off tails were needed for subsequent blood collection, which meant a less injury but a more convenient operation to a mouse. In contrast, previous CMS methods often collected blood samples from other sites, such as the tail vein [14, 15], saphenous vein [16], retro-orbital [17], and submandibular [18]. These CMS methods depend on the vein puncture technique, which required skillful technology, while repeated puncture during series of sampling usually leads to hematomas [19, 20]. In addition, unskillful puncture technique or hematomas often result in the deviation of sampling amount and time point from the plan and always lead to the poor quality of pharmacokinetic data.

Besides CMS, there are other similar microsampling techniques, such as DBS and VAMS. DBS is the collection of microvolumetric whole blood with filter paper [21]. However, DBS results can be influenced by several factors, and the most important one is the hematocrit and the homogeneity of the blood [22]. The hematocrit determines the viscosity of the blood and affects the diffusion of spotted blood on filter paper. This could result in blood volume fluctuations and thus bring bias in the concentration analysis [23]. The nonhomogeneity of DBS samples also affects the accuracy of the quantification [10]. As a consequence, the DBS technology cannot be a general alternative for sampling in pharmacokinetic studies [24]. As for VAMS, it involves microvolumetric absorption and the subsequent drying of the liquid sample in a porous tip that can collect 10–20 *μ*L blood for storage in cartridges, clamshells, or 96-well racks [25–27]. VAMS has been extensively used for pharmacokinetic studies and therapeutic drug monitor [28, 29]. However, the cost of the special devices is high because of a large number is needed in pharmacokinetic studies. Compared with the abovementioned sampling technologies, CMS is suitable to collect a series of blood samples with minimal damage, which provides credible samples for subsequent quantitative analysis.

### 3.2. HPLC-MS/MS

The developed HPLC-MS/MS was found to be sensitive, accurate, and repeatable for the determination of trans-resveratrol and its main metabolites.

#### 3.2.1. Specificity

**Figure 3:**
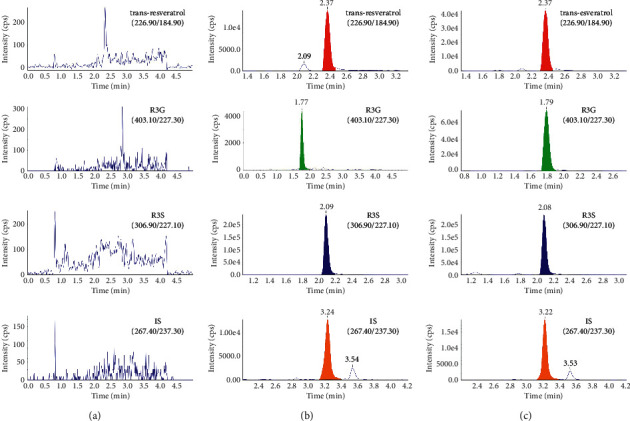
Typical multiple-reaction monitoring chromatograms of (a) a blank mouse blood; (b) a blank mouse blood sample spiked with trans-resveratrol, R3G, R3S, and IS; and (c) a blood sample 1 h after the intragastric administration of 150 mg/kg trans-resveratrol.

High selectivity was achieved by using HPLC-MS/MS to monitor only the ions derived from the analytes of interest. The typical MRM chromatograms of a blank blood sample; a spiked blood sample with trans-resveratrol, R3G, R3S, and IS; and a blood sample taken 1 h after the intragastric administration of 150 mg/kg trans-resveratrol to a mouse are shown in Figures 3(a)–3(c). No interference from any endogenous substance was observed at the retention times of any of the analytes. The retention times were approximately 2.37 min, 2.07 min, 1.79 min, and 3.22 min for the trans-resveratrol, R3G, R3S, and IS, respectively.

#### 3.2.2. Linearity and Lower Limit of Quantification

Linearity was assessed by seven-level calibration curves in the mouse blood on three consecutive days. Calibration curves were obtained in the range of 2.24–223.5 ng/mL for the trans-resveratrol and 1–200 ng/mL for the R3G and R3S. The mean linear regression equation for the calibration curve was *y* = 0.000172*x* + 0.00794 for the trans-resveratrol; *y* = 0.002*x* − 0.000516 for the R3G, and *y* = 0.0538*x* − 0.0147 for the R3S, where *y* was the concentration of the analyte and *x* was the peak area ratio of the analyte to the IS. The calibration curves exhibited good linearity with a mean ± SD correlation coefficient (*r*^2^) of 0.9908 for the trans-resveratrol, 0.994 for the R3G, and 0.9937 for the R3S. The LLOQ in the mouse blood was found to be 2.24 ng/mL for the trans-resveratrol and 1 ng/mL for the R3G and R3S. This met the requirements for pharmacokinetic studies.

#### 3.2.3. Accuracy and Precision

The intra- and interbatch precision and accuracy results for the determination of the trans-resveratrol, R3G, and R3S QC samples are presented in Table 2. For the devised assay, the relative standard deviation of the precision for the analytes was 3.0–10.9% for the intrabatch samples and 6.07–12.22% for the interbatch samples. Intrabatch accuracy was 90.3–112.6% and interbatch accuracy was 91.57–106.83% for all the analytes. The results met the FDA guidelines for the bioanalytical validation of precision and accuracy.

#### 3.2.4. Matrix Effect and Recovery

The matrix effect of the method was evaluated by analyzing the QC samples at low and high concentrations. The matrix effects were 103.37 ± 14.08% and 88.32 ± 4.75% for the trans-resveratrol, 105.32 ± 12.82% and 100.3 ± 4.46% for the R3G, and 97.95 ± 2.9% and 95.36 ± 2.95% for the R3S. This indicated that the ion suppression or enhancement of the analytes and the endogenous interference could be ignored. The details of the results are presented in Table 3.

The extraction recovery of the trans-resveratrol from the QC samples was 83.82 ± 8.12%, 79.97 ± 4.3%, and 79.66 ± 2.1% at three concentration levels (5.59, 22.35, and 111.75 ng/mL, respectively; *n* = 6). The extraction recovery of the R3G from the QC samples was 89.28 ± 6.42%, 83.54 ± 8.38%, and 83.38 ± 2.04% at three concentration levels (5, 20, and 100 ng/mL, respectively; *n* = 6). The extraction recovery of the R3S from the QC samples was 89.46 ± 5.21%, 89.52 ± 1.52%, and 85.19 ± 2.98% at three concentration levels (5, 20, and 100 ng/mL, respectively; *n* = 6). In addition, the extraction recovery of the IS was 99.94 ± 4.78%. The consistency in the recovery of the trans-resveratrol, R3G, R3S, and IS supported the suitability of the extraction procedure for routine sample analysis. The detailed results are presented in Table 3.

#### 3.2.5. Stability

The percent CV for the peak area between the stocked solutions (in methanol at 4°C for 30 days) and the freshly prepared solutions was 8.54% for the trans-resveratrol, 6% for the R3G, 6.28% for the R3S, and 6.62% for the IS. This indicated that the stock solutions were stable at 4°C for at least 30 days. The stability results under specific storage conditions are summarized in Table 4. No chemical or biological degradation of the trans-resveratrol, R3G, or R3S was observed during sample storage, preparation, or analysis. All the samples exhibited 85–115% recovery after the various stability tests. This indicated that the method was appropriate for routine analysis.

In spite of sufficient biological sample size achieved by CMS, the blood sample volume at each point was very small. This has created a high demand for improvements in the sensitivity of the detection technology, while the rapid development of mass spectrometry technologies, especially the new generation of HPLC-MS/MS, has facilitated the achievement of trace detection for most compounds. In present HPLC-MS/MS method, the sensitivity of trans-resveratrol, R3G, and R3S were 2.24, 1.0, and 1.0 ng/mL, respectively, and it only costs 10 *μ*L of blood sample volume with a simple protein precipitation (PP). PP sample preparation is simpler and more economical than solid-phase extraction (SPE) and liquid-liquid extraction (LLE). SPE requires special cartridges, which are somewhat expensive, not only because of their cost but also their non-reusability. In addition, the preparation procedure, which is complicated and time-consuming, offers no benefits for the extraction of the unstable compounds such as trans-resveratrol and its metabolites [30]. LLE is another popular method for the sample preparation. However, LLE needs a great deal of time for extractant evaporation; thus, it is also ineffective for unstable compounds [31].

### 3.3. Application of the Joint Technology

**Figure 4:**
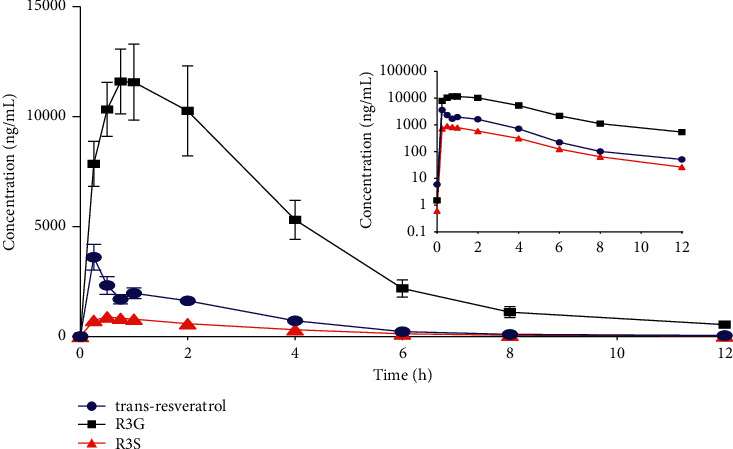
Mean blood concentration-time curves of trans-resveratrol, trans-resveratrol-3-o-*β*-glucuronide (R3G), and trans-resveratrol-3-sulfate salt (R3S) in blood from six mice after the intragastric administration of 150 mg/kg trans-resveratrol.

The present joint technology was successfully applied to the pharmacokinetic study of trans-resveratrol and its main metabolites in mice. The modified CMS technique guaranteed the quality of sample collection for the subsequent determination of targeted analytes by HPLC-MS/MS. The concentrations of trans-resveratrol and its metabolites were determined up to 12 h for all the mice with HPLC-MS/MS. The profiles of the mean blood concentrations of trans-resveratrol and its metabolites are presented in Figure 4. The main pharmacokinetic parameters for trans-resveratrol and its metabolites are presented in Table 5.

**Figure 5:**
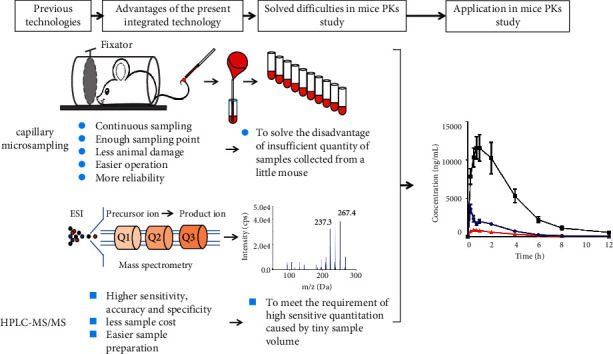
The advantages of a joint technology combined the CMS with HPLC-MS/MS in mice pharmacokinetic studies.

The pharmacokinetic parameters are comparable with those reported in previous studies [12, 32], thereby indicating the reliability of this joint technology for the pharmacokinetic study of trans-resveratrol and its metabolites in mice. For example, Bohmdorfer et al. found the *T*_max_ of trans-resveratrol, R3G, and R3S to be 0.5 h [33]. Menet et al. reported concentrations of trans-resveratrol, R3G, and R3S to be 5628.40, 61391.45, and 4432.49 ng/mL, respectively, after administration of 150 mg/kg trans-resveratrol to mice [32]. Although these results are similar to ours, the number of specimens used to calculate the pharmacokinetic parameters per mouse is very small. Thus, the result could not reflect the precise pharmacokinetic characteristics of the target compounds. In contrast, our study used 10 time-points blood samples per mouse, and the pharmacokinetic parameters obtained are relatively more accurate and reliable. The present joint technology displays obvious advantages including less animal damage, easier sample preparation, and improved reliability (Figure 5).

## 4. Conclusions

A joint technology combining the advantages of CMS and HPLC-MS/MS was successfully established and applied to the pharmacokinetic study of trans-resveratrol and its main metabolites in mice. It simultaneously satisfied the requirements of high quality of the sample and high sensitivity of detection, thus provided a more effective option for future pharmacokinetic studies in mice.

## Figures and Tables

**Table 1:** Comparison of the actual blood collection time and the appointed blood collection time in six mice.

	Appointed time (min)									
	0	15	30	45	60	120	240	360	480	720
Average	0	14.83	30.33	45.17	60.58	120.92	240.42	360.00	482.50	720.50
SD	0	0.55	1.11	1.14	1.26	2.02	0.76	0.58	4.05	1.85
CV (%)	-	3.73	3.64	2.53	2.07	1.67	0.32	0.16	0.84	0.26

Data of average, SD, and CV were calculated from the actual blood collection time per time-point collection in six mice. Abbreviations. SD, standard deviation; CV, variable coefficient; -, not calculated.

**Table 2:** Intrabatch (*n* = 5) and interbatch (*n* = 15) accuracy and precision of trans-resveratrol, R3S, and R3G in mice blood at four QC levels.

Analytes	Nominal concentration (ng/mL)	Intrabatch (*n* = 5)			Interbatch (*n* = 15)		
		Measured concentration (ng/mL)	Precision (%)	Accuracy (%)	Measured concentration (ng/mL)	Precision (%)	Accuracy (%)
Trans-resveratrol	2.24	2.30 ± 0.25	10.91	103.23	2.31 ± 0.20	8.81	103.71
	5.59	5.93 ± 0.56	9.45	106	5.52 ± 0.50	9.11	98.65
	22.35	25.24 ± 0.47	1.87	112.6	23.69 ± 2.13	9.00	105.69
	111.75	108 ± 4.52	4.18	96.28	107.34 ± 9.02	8.40	95.79

R3G	1.00	1.07 ± 0.15	14.00	106.9	1.03 ± 0.13	12.22	103.44
	5.00	5.25 ± 0.30	6.00	104.9	5.33 ± 0.32	6.07	106.46
	20.00	18.1 ± 0.91	5.00	90.48	18.31 ± 1.25	6.86	91.57
	100.00	90.7 ± 4.67	5.00	90.7	96.21 ± 7.74	8.04	96.21

R3S	1.00	1.02 ± 0.08	8.11	101.50	0.96 ± 0.09	9.38	96.05
	5.00	5.19 ± 0.22	4.29	103.87	5.34 ± 0.37	6.97	106.83
	20.00	18.04 ± 0.61	3.00	90.30	18.67 ± 1.65	8.84	93.33
	100.00	103.07 ± 9.13	8.86	103.07	100.73 ± 10.96	10.88	100.73

Data of measured concentration represented as mean ± SD. R3G, trans-resveratrol-3-o-*β*-glucuronide; R3S, trans-resveratrol-3-sulfate salt.

**Table 3:** Matrix effect and recovery of trans-resveratrol, R3G, R3S, and diethylstilbestrol (IS).

Analytes	Matrix effect		Recovery (%)		
	LQC	HQC	LQC	MQC	HQC
Trans-resveratrol	103.37 ± 14.08 (13.62)	88.32 ± 4.75 (5.38)	83.82 ± 8.12 (9.69)	79.97 ± 4.30 (5.38)	79.66 ± 2.10 (2.64)
R3G	105.32 ± 12.82 (12.17)	100.30 ± 4.46 (4.45)	89.28 ± 6.42 (7.20)	83.54 ± 8.38 (10.03)	83.38 ± 2.04 (2.45)
R3S	97.95 ± 2.90 (2.96)	95.36 ± 2.95 (3.10)	89.46 ± 5.21 (5.82)	89.52 ± 1.52 (1.70)	85.19 ± 2.98 (3.49)
Diethylstilbestrol	96.41 ± 10.09 (10.46)		99.94 ± 4.78 (4.78)		

Data are represented as mean ± SD (RSD%). R3G, trans-resveratrol-3-o-*β*-glucuronide; R3S, trans-resveratrol-3-sulfate salt.

**Table 4:** Stability of trans-resveratrol, R3G, R3S, and IS under several conditions.

Analytes	Stability under various conditions (mean (RSD%), % of nominal concentration) (*n* = 4)		
	Low QCs	Middle QCs	High QCs
*Short-term stability (ambient for 2 h)*			
Trans-resveratrol	101.32 (8.78)	101.62 (10.05)	93.15 (4.83)
R3G	106.18 (9.38)	96.80 (3.46)	97.83 (10.35)
R3S	101.13 (5.94)	92.85 (4.23)	93.35 (8.98)

*Light stability (for 2 h)*			
Trans-resveratrol	108.25 (4.85)	99.00 (10.88)	90.70 (4.11)
R3G	97.30 (6.92)	92.43 (3.80)	95.77 (1.79)
R3S	95.75 (5.50)	90.78 (4.37)	99.33 (1.61)

*Autosampler stability (for 12 h)*			
Trans-resveratrol	91.05 (4.68)	105.60 (7.77)	93.20 (9.52)
R3G	91.52 (4.51)	89.34 (5.60)	89.47 (9.19)
R3S	99.02 (5.37)	92.28 (5.53)	94.22 (8.67)

*Freeze (−80°C)-thaw (25°C) for three cycles (12 h for one circle)*			
Trans-resveratrol	98.63 (13.20)	88.83 (2.67)	110.40 (4.13)
R3G	91.33 (8.09)	88.18 (2.48)	87.18 (1.97)
R3S	94.76 (4.79)	93.18 (4.56)	98.92 (6.91)

*Long-term stability(−80°C for 30 days)*			
Trans-resveratrol	106.44 (9.98)	106.74 (7.64)	95.84 (6.50)
R3G	102.16 (10.95)	97.42 (9.88)	95.48 (5.21)
R3S	101.45 (9.85)	94.45 (5.10)	87.23 (2.60)

*Stocked solutions stability (4°C for 30 days)*			
Trans-resveratrol	92.79 (1.77)		
R3G	90.37 (3.10)		
R3S	94.00 (2.89)		
IS	104.09 (8.96)		

R3G, trans-resveratrol-3-o-*β*-glucuronide; R3S, trans-resveratrol-3-sulfate salt; IS, internal standard; QCs, quality control samples.

**Table 5:** Pharmacokinetic parameters of trans-resveratrol and its metabolites in mice after a single intragastric administration of trans-resveratrol at a dosage of 150 mg kg^−1^ (*n* = 6).

Analytes	Parameters (mean ± SD)				
	AUC_(0–12)_ (ng·h mL^−1^)	AUC_(0–∞)_ (ng·h mL^−1^)	*C*_max_ (ng mL^−1^)	*T*_max_ (h)	t_1/2_ (h)
Trans-resveratrol	8335.03 ± 4980.70	8463.70 ± 5000.20	5969.17 ± 3838.91	0.5 ± 0.282	1.90 ± 1.23
R3G	51027.06 ± 30317.97	53016.13 ± 30991.44	13799.17 ± 7070.70	0.77 ± 0.46	2.24 ± 1.04
R3S	3083.44 ± 1625.43	3163.12 ± 1624.85	971.75 ± 485.22	0.60 ± 0.27	2.08 ± 0.86

R3G, trans-resveratrol-3-o-*β*-glucuronide; R3S, trans-resveratrol-3-sulfate salt; AUC_(0-12)_, area under the plasma concentration-time curve from 0 to 12 hours; AUC_(0–∞)_, area under the plasma concentration-time curve from 0 to infinity hours; *T*_max_, time to peak concentration; *C*_max_, maximal concentration; *t*_1/2_, eliminate half-life.

## Data Availability

All data generated or analyzed during this study are included in this published article.
